# Identity-Based Behavioral Economic Incentives Increased PrEP Adherence for Transgender and Nonbinary Adults in a Trans-Led PrEP Navigation Program

**DOI:** 10.1007/s10461-026-05206-2

**Published:** 2026-07-14

**Authors:** Kimberly Ling Murtaugh, Alex R. Dopp, Carrie L. Nacht, Risa Flynn, Chloe Opalo, Erik D. Storholm

**Affiliations:** 1 Department of Public Policy, Luskin School of Public Affairs, University of California, Los Angeles, Los Angeles, CA, USA; 2 Center for HIV Identification, Prevention, and Treatment Services (CHIPTS), University of California, Los Angeles, CA, USA; 3 RAND, Santa Monica, CA, USA; 4 School of Public Health, San Diego State University, 5500 Campanile Drive, Hepner Hall 114E, San Diego, CA 92182-4162, USA; 5 Herbert Wertheim School of Public Health and Human Longevity Science, University of California San Diego, La Jolla, CA, USA; 6 Los Angeles LGBT Center, Los Angeles, CA, USA; 7 Trans Wellness Center, Los Angeles, CA, USA

**Keywords:** Behavioral Economics, Identity Economics, PrEP, HIV Prevention, Transgender community, Health equity

## Abstract

Tailored supports are needed to maximize PrEP engagement among transgender and nonbinary (TGNB) adults, a population disproportionately impacted by HIV with low PrEP use, due to structural and economic barriers, particularly among TGNB individuals of color. This study piloted the integration of identity-based behavioral economic incentives, which use the science of decision-making to encourage beneficial behaviors, within an ongoing community-led, multi-component PrEP navigation program called *PrEP Well*. Prizes were selected through TGNB community input, were TGNB-affirming and specific to community needs, and included items sourced from TGNB-owned businesses; participants earned prize-wheel spins for PrEP-related steps. Among 108 TGNB adults at a trans-led Los Angeles community center (2022–2023; 36 enrolled before and 72 after incentives began), we conducted a non-randomized pilot trial assessing the acceptability, feasibility, and preliminary effects of incorporating these incentives into *PrEP Well*. Pilot results showed that, compared to standard *PrEP Well*, the addition of identity-based behavioral economic incentives was seen as feasible and acceptable, and produced a moderate effect on PrEP adherence at 90 days post-enrollment (78.6% vs. 56.3%, *X*^*2*^_(1, 108)_ = 4.38, *p* < .05, φ = 0.20). Dose-response models indicated that each additional spin earned through the incentive program further increased the odds of PrEP adherence at 90 days by about 4.7-fold (95% confidence interval [2.34, 9.32], *B* = 1.54, *p* < .001). This improvement was achieved at a modest additional cost of approximately $51 per participant. Integrating behavioral economic incentives into community-based, identity-affirming service delivery models may be a promising, cost-effective approach for enhancing PrEP engagement and improving HIV prevention outcomes among TGNB adults.

## Introduction

HIV prevalence among transgender and nonbinary (TGNB) adults is disproportionately high in the U.S. Nationally, HIV prevalence among transgender women ranges from 14% to 28% [1–3], exceeding the prevalence among cisgender sexual minority men [4]. Data also suggest that HIV rates are increasing among transgender men [5]. HIV rates for nonbinary adults remain largely unknown, but are presumed elevated due to shared vulnerabilities. A wide range of multi-level factors contribute to the increased HIV risk among TGNB individuals, including substance use, mental health conditions, sexually transmitted infections, incarceration, un/underemployment, poverty, reliance on sex work, stigma, discrimination, transphobia, racism, immigration status, language barriers, limited educational attainment, violence, sexual assault, housing instability, and sociopolitical attacks on TGNB existence [1, 6].

Pre-exposure prophylaxis (PrEP) is an evidence-based intervention proven to prevent HIV infection. However, PrEP uptake remains disproportionately low among TGNB people, especially those of color, due to the same social, structural, and economic factors associated with increased rates of HIV in this population [7, 8]. Tailored supports are urgently needed to improve PrEP uptake and persistence among TGNB adults [7–11]. Standard HIV prevention approaches have led to much greater PrEP uptake among cisgender sexual minority men compared to TGNB people, and among white TGNB people compared to TGNB people of color, underscoring the need for solutions that address these specific inequities rather than simply extending existing models [12, 13].

To address these inequities in PrEP uptake, our community-academic collaborative research team piloted the addition of behavioral economic incentives to encourage engagement along the PrEP care continuum with our *PrEP Well* navigation program, which operates in a TGNB community center in Los Angeles. Behavioral economics is an interdisciplinary field that integrates economics, psychology, and other social sciences to design strategies that increase engagement in beneficial behaviors (e.g., PrEP use) [14, 15]. To create behavioral economic strategies for this project, we specifically applied the principles of identity economics, which posit that beneficial actions are most effectively motivated by a combination of incentives with extrinsic value (e.g., monetary prizes) and intrinsic value (e.g., alignment with an individual’s identity or self-concept) [15–18].

Past studies have successfully used financial incentives to encourage positive health behaviors (e.g., medication adherence), leveraging the broad motivational value of money [19–21]. Incentives have been shown to increase a wide variety of desired health behaviors like smoking cessation, weight loss, increased exercise, and increased adherence to medications for chronic conditions [19–21]. Given the established benefits of financial incentives, *PrEP Well* clients receive them as a standard program element for each visit completed with the program navigator. Some studies have shown evidence of PrEP uptake and adherence in response to financial incentives, with mixed results on prizes versus lotter-based incentives [22–24]. In the current study, we used several identity-based strategies based in behavioral economic approaches to motivate PrEP engagement and adherence beyond the standard program financial incentives, with both the selection of target behaviors and the design of rewards tailored to the TGNB community.

The use of tangible prize-based incentives can be motivating for several reasons, including access to goods that participants might not otherwise purchase, increased task engagement, and the enjoyment associated with selecting or winning a prize [25]. Studies have tested different incentive schedules, including varying the likelihood of winning prizes of different value. Prize-based approaches have been shown to be at least as effective as cash or vouchers in supporting health behavior change, even when the value of the prizes is much less than the voucher value [26, 27]. Introducing chance-based methods (e.g., a prize wheel) also increases engagement and positive associations with the task compared to guaranteed payments [28]. Moreover, providing prizes immediately offers associative reinforcement and has been related to achieving long-term health goals [29].

Identity economics expands the view of individuals beyond purely being rational actors, recognizing that their motivations and behaviors are shaped by emotional, cognitive, and social influences tied to their identity and self-concept (i.e., intrinsic motivation). Individuals make relative judgments of value for a given behavior and its outcomes, based on their perceptions about the extent to which people like them would engage in that behavior for those anticipated outcomes [16–18]. For those in a social group facing significant inequities, such as TGNB people, behavioral economic incentives that respond to community-specific needs, support community-owned businesses, and uniquely relate to aspects of their shared identity may have stronger effects than financial incentives alone.

In sum, our community-led approach aimed to respond to the needs and motivations of *PrEP Well* participants by providing tailored incentives for PrEP engagement that were identity-based and built upon standard financial incentives. This paper reports the findings from our pilot evaluation of the identity-based behavioral economic incentives, which includes our assessment of the incentives’ acceptability and feasibility as well as preliminary effects on engagement in the PrEP care continuum.

## Method

### Study Design

From August 1, 2022 through December 31, 2023, we conducted a non-randomized, two-group trial to evaluate the impact of identity-based behavioral economic incentives within the trans-led *PrEP Well* navigation program. *PrEP Well*, operated at a TGNB-led community center in Los Angeles, provides navigation and support services to promote PrEP uptake among TGNB adults [12]. A nonrandomized phased design was chosen in response to community partner concerns that randomization could create perceptions of unfairness and mistrust within the center’s tightly knit client social networks. To strengthen inference, we employed a convergent mixed-methods approach that integrated quantitative outcomes, qualitative interviews, and implementation/process data. We compared (1) the standard *PrEP Well* program with visit-based financial incentives across the initial 37 weeks of enrollment and (2) the *PrEP Well* program plus identity-based behavioral economic incentives during the subsequent 37 weeks after those incentives were introduced. This phased structure ensured comparability by drawing evaluation data from the first 37 weeks of each condition [30].

### Study Procedures

To minimize participant burden and optimize resources, we integrated study procedures into the standard *PrEP Well* workflow whenever possible. *PrEP Well* study procedures have been previously published elsewhere [12]. All enrollments and services were coordinated by the *PrEP Well* navigator, a trained community member. Eligible participants were TGNB adults (≥ 18 years) who were HIV-negative, not enrolled in another PrEP intervention or study, and willing to undergo PrEP monitoring via point-of-care urine and dried blood spot testing. Recruitment strategies included outreach to clients at the community center, referrals from other center programs, and online or in-person engagement at community events. We encouraged participation from TGNB adults of all backgrounds, with a particular focus on TGNB persons of color due to inequities in PrEP uptake.

Clients who tested HIV-negative and met eligibility criteria were informed about the *PrEP Well* program and invited to participate. At enrollment, staff emphasized that participation was voluntary and would not affect access to PrEP or other services at the center. Those who agreed provided informed consent and completed a Qualtrics-administered baseline survey to collect sociodemographic characteristics and measures for monitoring throughout the program.

*PrEP Well* services included outreach and education, HIV testing, benefits navigation, appointment scheduling, PrEP prescription visits, pharmacy assistance, and follow-up. Participants were scheduled for a 30-day follow-up visit to assess PrEP uptake and provide adherence support, followed by a 90-day appointment to evaluate ongoing PrEP use and address any barriers. Additional data, including PrEP engagement outcomes (e.g., uptake, sustained use, and adherence) and, at the 90-day visit, measures of acceptability and feasibility, were collected via Qualtrics surveys at the 30- and 90-day visits, and participants could contact the *PrEP Well* navigator between visits for support. All incentives (including, when relevant, the identity-based prizes) were administered during the enrollment, 30-day, and 90-day visits. Incentive details were outlined in the consent form.

### Intervention Conditions

All participants received support from the overall *PrEP Well* program, as described previously [12]. The key comparison for our pilot study was between two conditions: (1) financial incentives only (standard condition) and (2) financial incentives plus identity-based incentives grounded in identity economics (identity-based behavioral economic enhancement condition).

### Standard PrEP Well Program (Financial Incentives Only)

Although *PrEP Well* visits were brief and focused on navigation for medical services, participants received a financial incentive for completing research surveys at each time point. Specifically, participants received $50 at enrollment, $75 at the 30-day visit, and $100 at the 90-day visit. These payments covered the effort and discomfort involved in evaluation activities (e.g., PrEP level testing) and helped maximize the immediate value of *PrEP Well* participation for economically vulnerable clients. This condition was implemented during the first 37 weeks of enrollment.

### Identity-Based Behavioral Economic Enhancements (Financial + Identity-Based Incentives, “the wheel”)

Beginning approximately nine months into *PrEP Well* implementation, we added identity-based incentives to the program visit schedule. Specifically, newly enrolled participants were offered additional prize-based rewards available through spins on a prize wheel.

### Prize Earning Behaviors

Typically, incentives for PrEP adherence are tied to lab-verified medication use measured at predetermined intervals [22]. In *PrEP Well*, we expanded this approach by also rewarding intermediate steps in the PrEP care continuum that often present barriers for TGNB individuals [31]. For example, participants earned a reward for attending an initial appointment with a PrEP provider. In previous interviews with community members, we heard how challenging it can be to find a PrEP provider who offers gender-affirming care and has appointment availability [32]. By reinforcing these sub-steps, we aimed to acknowledge the significant effort involved in navigating fragmented care systems. Breaking down tasks in this way can increase success at completing the overall behaviors, provide an enhanced sense of self-efficacy, and give transparent positive reinforcement [33].

Spins on the prize wheel were awarded for behaviors that represented important steps in the PrEP care continuum, such as attending a *PrEP Well* information session, attending initial PrEP provider appointment, completing lab/STI testing, or providing a urine test showing PrEP use. Table 1 lists all eligible behaviors. This list was shared with participants during their initial onboarding session and was revisited at each visit. Participants were given a small checklist card outlining the eligible behaviors and received ongoing reinforcement from the *PrEP Well* navigator (e.g., “great job filling your prescription, you have earned a spin at your next visit”).

Behaviors that earned spins were selected to break down the multi-step engagement process, reinforce intermediate milestones, and reflect community-specific challenges such as difficulty obtaining appointments with gender-affirming providers. In cases where participants demonstrated significant effort but were unable to complete a behavior due to circumstances beyond their control, the navigator could award one discretionary spin to reinforce effort. Participants could earn a maximum of three spins per visit.

### Prize Structure and Implementation

Prizes were available on site, and participants spun the wheel, selected, and received their prizes immediately at enrollment, 30-day, and 90-day visits alongside the standard financial incentives. We used a probability-based earning structure with three prize tiers, color-coded to reflect the transgender pride flag. The tiers varied by prize value and likelihood of landing on that color. The wheel had 24 slots: 16 pink, 7 blue, and 1 silver. Market values were: pink tier ($5–15), blue tier ($20–50), and silver tier ($70–100) (with actual costs reduced through bulk purchasing and discounts).

Each spin gave participants the chance to win a prize within the corresponding tier, and earning more spins increased the odds of receiving higher-value items. The *PrEP Well* navigator documented qualifying behaviors at each visit to ensure transparent and consistent reward allocation.

### Prize Selection of TGNB Affirming Items and Engagement of TGNB-Owned Businesses

Our team partnered with community members and *PrEP Well* clients to identify prizes that affirmed TGNB identities. Whenever possible, prizes were sourced from TGNB-owned businesses, so participant engagement simultaneously supported other TGNB community members. Businesses ranged from highly established Stealth & Co. (producer of travel injection supply kits featured on the television show, Shark Tank) to local home-based businesses (e.g., tucker panties). We also partnered with a TGNB artist to create a “My Health × My Terms” exclusive *PrEP Well* design. Although sourcing items from TGNB-owned businesses was more time-consuming and costly than purchasing from large retailers, a balanced mix of items helped manage costs.

Based on focus groups and participant feedback, we compiled an initial prize “store,” which was restocked over time and updated with new items to maintain interest. A prize “lookbook” displayed photos of available items, their tier, and a badge indicating TGNB-owned sourcing when applicable. Participants could review the lookbook, select prizes, and receive them immediately during their visit.

These enhancements combined multiple behavioral economic strategies to reinforce desired PrEP engagement behaviors in a predictable, identity-affirming way that honored the experience of community members and reduced HIV risk. Beyond traditional behavioral economic principles, our approach incorporated social norm strategies and identity economics to reflect the central role of community in decision-making among TGNB clients and center staff. Furthermore, the design of these enhancements was guided by equitable research practices that centered community feedback. For example, we first demonstrated the wheel and all prizes with members of our community advisory board and incorporated their feedback. In aligning incentives with TGNB identities, we aimed to enhance engagement at every step of the PrEP care continuum.

### Implementation Frameworks

The design and evaluation of this pilot were guided by the EPIS (Exploration, Preparation, Implementation, Sustainment) framework [34], which outlines the process of implementing novel practices in community settings. During the Exploration phase, formative interviews and focus groups with TGNB community members identified barriers to PrEP engagement and shaped the design of acceptable incentive strategies. In the Preparation phase, we collaborated with TGNB participants and community partners to select prizes, develop the “lookbook,” and establish sourcing partnerships with TGNB-owned businesses. The Implementation phase focused on integrating the prize wheel and incentive delivery into existing *PrEP Well* workflows at the Trans Wellness Center (TWC), including navigator training and development of inventory and distribution systems. Transition into the long-term Sustainment phase was informed by examining budget impact, prize selection trends, and participant/staff perceptions of feasibility to inform scalability.

We also made selective use of elements from the RE-AIM (Reach, Effectiveness, Adoption, Implementation, Maintenance) framework [35] to measure outcomes most relevant to pilot testing. These outcomes are described next under Measures.

### Measures

Our evaluation measures focused on three key RE-AIM outcome domains: effectiveness, implementation (including feasibility and acceptability), and maintenance of the two *PrEP Well* conditions. *Effectiveness* was defined as participants’ engagement along the PrEP care continuum (e.g., linkage to prescription, uptake, sustained use, and adherence) [36]. Acceptability and feasibility are related concepts within the *Implementation* domain: acceptability refers to whether participants and staff perceive that a service (such as the incentives) should be offered, while feasibility reflects whether the service can be practically delivered within routine program operations given the available resources and competing needs [37]. We assessed acceptability and feasibility from the perspective of participants and center staff. We also examined the budget impact of identity-based incentives (i.e., prizes) as a feasibility indicator, which further relates to *Maintenance* (i.e., potential sustainability based on costs and staff burden).

### Received PrEP Prescription

Participants attended a dedicated PrEP visit with a healthcare provider, during which laboratory tests were conducted and a prescription was written. PrEP visit attendance and prescription receipt were reported by participants and documented at the 30-day follow-up. The peer navigator cross-checked against the electronic medical record to ensure accuracy. To encourage flexibility and real-world applicability, participants could choose to see a provider onsite at the center or off-site based on availability and client preferences, and this information was noted in the study record.

### PrEP Uptake

Uptake of oral PrEP was verified at 30-day follow-up using SureQuick^®^, a point-of-care diagnostic test used to detect Tenofovir (TFV) in urine. This provided rapid, reliable confirmation that PrEP had been taken. Participants reporting uptake of long-acting injectable cabotegravir were verified to have received injection via their electronic medical records.

### Sustained PrEP Use

Sustained daily oral PrEP use at 90-day follow-up was also verified with SureQuick^®^. Participants reporting continued use of long-acting injectable cabotegravir were verified to have received an additional injection prior to their 90-day follow-up visit via their electronic medical records.

### PrEP Adherence

Adherence to daily oral PrEP was assessed at the 90-day follow-up using dried blood spot (DBS) assays, which quantify long-term intracellular TFV-DP levels in the blood. DBS concentrations consistent with 4 or more doses/week were classified as adherent. Participants verified to be on long-acting injectable cabotegravir were classified as adherent if their 90-day follow-up visit occurred within 60 days of their most recent injection.

### Earned Spins and Prizes

Participants could earn up to three spins per visit at baseline, 30, and 90 days. Although there were 12 total eligible spin-earning behaviors across the three visits (see Table 1), the maximum spins per participant was 9. At each visit, the number of spins earned, prize tier, and the prize(s) selected were recorded.

### Acceptability and Feasibility

Acceptability was assessed through qualitative exit interviews and brief surveys with participants conducted at or after the 90-day visit. Participants were asked about their satisfaction with the incentives, whether they felt the incentives were appropriate and motivating, and if they would recommend that the program continue offering them. Acceptability and feasibility were assessed by surveys administered to the center staff, which inquired about perceptions of the program, including if staff felt the program was a good fit for the center, and would be easily maintained at the center.

### Budget Impact

Budget impact was assessed by tracking the direct costs of purchasing and maintaining an inventory of identity-based prizes in comparison to the financial incentives provided in the standard condition. We documented the per-participant cost of incentives, bulk-purchasing strategies, and sourcing from TGNB-owned businesses to evaluate sustainability and scalability.

### Data Analysis

#### Quantitative Analysis

Quantitative data were analyzed to determine differences in effectiveness outcomes (i.e., received PrEP prescription, PrEP uptake, sustained PrEP use, and PrEP adherence). We used descriptive statistics to summarize the demographic characteristics of the sample, including age, gender identity, race/ethnicity, and relationship status. To test for differences in demographic factors by study conditions, we conducted bivariate analyses using Chi-square tests for categorical variables and t-tests for continuous variables [38]. We then also conducted Chi-square tests to compare differences in participant rates in achieving each program outcome between the two intervention conditions. The effect size for each Chi-square test was then calculated using the phi coefficient (φ) [39].

To evaluate whether program engagement demonstrated a dose–response relationship with PrEP adherence, we conducted a series of binary logistic regression models with biochemical evidence of PrEP adherence at the 90-day follow-up as the dependent variable. Separate models examined as predictors (1) the number of spins earned by the 30-day visit and (2) total spins accumulated across the 90-day study period. Model fit was evaluated using the Hosmer-Lemeshow goodness-of-fit test, and explained variance was estimated using Nagelkerke *R*^*2*^. Odds ratios (ORs) and 95% confidence intervals (CIs) were reported to describe the magnitude and direction of associations. All analyses were conducted using IBM SPSS Statistics (version 31.0) [40]. The statistical significance level was set at *p* < .05.

#### Qualitative Analysis

Exit interviews with participants and staff were audio-recorded, transcribed, translated to English if necessary, and analyzed using inductive thematic analysis [41]. Three researchers read through and open-coded a subset of five transcripts representing diversity in sociodemographic attributes, study completion rates, and PrEP adherence levels. After open coding, the researchers developed an initial codebook. The codebook was iteratively tested and revised by two researchers, who double-coded interview transcripts and discussed any discrepancies or disagreements with a third researcher. After reaching an acceptable inter-rater reliability (κ > 0.80), the remaining transcripts were coded. We then identified key themes to capture experiences with the prize wheel, perceived motivational value of incentives, and reflections on TGNB-affirming prize selection. NVivo (version 14) was used for coding and analysis [42].

#### Budget Impact Analysis

We compiled prize cost data through receipts, invoices, and inventory logs maintained by the PrEP Well navigator. Costs were summarized by total amount spent, average cost per participant, and average cost per prize distributed. To contextualize feasibility, we calculated unit costs relative to program duration and number of participants served.

#### Mixed-Methods Integration

We used a convergent mixed-methods approach to iteratively collect and compare quantitative outcomes with qualitative themes to generate a comprehensive understanding of program effects [43]. Quantitative findings on PrEP outcomes were interpreted alongside qualitative insights into participant and staff experiences, enabling us to assess not only whether the incentives improved engagement, but also how and why they were experienced as acceptable, feasible, and identity-affirming.

#### Ethical Considerations

The study received approval from the institutional review board of San Diego State University. All participants provided written informed consent prior to enrollment. We ensured confidentiality and anonymity of participant data, particularly regarding sensitive HIV status and PrEP adherence information. Study data were stored on secure, password-protected servers, and only de-identified data were used for analysis. Given the vulnerability of TGNB populations, additional safeguards included staff training in gender-affirming and trauma-informed research practices, and procedures to ensure that participation did not affect access to services at TWC.

## Results

Table 2 summarizes participant demographics, which were highly diverse in terms of age, gender identity, race/ethnicity, sexual orientation, and educational background. This diversity reflects the varied experiences and needs of the target population, supporting the generalizability of findings to TGNB communities. Statistical analyses, including independent t-tests and Chi-square tests, did not reveal any significant differences in participant sociodemographic characteristics between the two study conditions (behavioral economic incentive condition vs. standard *PrEP Well* condition) prior to the intervention. This baseline comparability strengthens the validity of subsequent findings.

### Implementation Acceptability and Feasibility

Participants in the behavioral economic incentive condition reported high acceptability of the intervention. The addition of prize-based incentives to the *PrEP Well* program was well received by participants as well as program staff. Exit interviews emphasized that the wheel was “fun,” evoked feelings of “childhood,” and made the program more enjoyable. Several participants noted the value of TGNB-affirming prizes, describing them as “things made by us, for us.”

“I love that the whole thing was trans-oriented, so it was very personable to my community. The colors, the theme, the prizes very thoughtfully picked out. The necklace, the jeans, the pillow. It was something very personal to the community that was in the right place and was executed beautifully. You did such a thoughtful job.” *− 36-year-old multi-racial trans woman, consistently adherent to PrEP.*

“[The wheel] makes you happy. That motivates you to stay in the program. That’s one of the things that motivates you to continue in the program. Because it’s something out of the ordinary, it’s different and then, it’s also fun, it’s nice.” *− 18-year-old Latinx/Hispanic nonbinary client, consistently adherent to PrEP.*

“The prize wheel is really cute. And I like your prizes. They’re just adorable and they’re like good quality prizes. It definitely just, you know, gives you another reason, another thing to look forward to for your research meetups.” *− 23-year-old Latinx/Hispanic trans man, consistently adherent to PrEP.*

Data from this pilot study also suggests that the behavioral economic incentive condition was feasible to implement within the community center context. Staff reported that administering the prize wheel and incentives fit within visit workflows, and participants appreciated receiving prizes immediately. Nine of 11 TWC staff surveyed (82%) agreed or strongly agreed that PrEP Well seemed easy to implement at the TWC, and 100% agreed or strongly agreed that PrEP Well was applicable, suitable, implementable, doable, possible, and a good match for the TWC.

“[The PrEP Well coordinator] is always very pleasant and I like her method of calling in with the patient to make sure they have more than a warm hand off…it is a LOT of patients she has connected to PrEP. I hope PrEP Well can expand so we can continue to lower new HIV infections rates.” − *TWC staff member*.

Nearly half of participants (35/72; 48.6%) selected at least one TGNB identity-based prize (e.g., tucker panties, artwork, or items sourced from TGNB-owned businesses). TGNB-affirming prizes were described in the exit interviews as particularly meaningful. One participant shared, “it was really cool to see trans-owned prizes or businesses offering prizes I think because it’s important, I think, to support each other economically,” *(31-year-old Latinx/Hispanic client, gender identified as “none”, consistently adherent to PrEP)*. Among participants who landed on a blue-tier spin that offered a choice between a solid-color fuzzy blanket and a Trans Pride flag blanket (n = 20), 14 chose the Trans Pride flag option compared to six who selected the solid color, demonstrating a clear preference for an identity-based prize. Subtler TGNB-themed prizes, such as crystal bracelets with Trans Pride flag colors, were also frequently chosen. One participant shared that they wore the trans flag colored bow tie and suspenders earned in the study at their wedding.

### Effectiveness

Table 3 illustrates that adherence to PrEP at 90 days was significantly higher among participants who received the behavioral economic incentives. A Chi-square analysis revealed that 78.6% of participants in the behavioral economic incentive condition were PrEP adherent at 90-day follow-up, compared to 56.3% of participants in the standard condition, *X*^*2*^(1, *N* = 108) = 4.38, *p* <.05, *φ* = 0.20. This moderate effect size suggests that the identity-based behavioral economic incentives meaningfully improved 90-day adherence rates. Results also indicated that the addition of incentives was associated with a greater number of participants enrolling in the program, receiving a PrEP prescription, and demonstrating PrEP uptake at the 30-day follow-up, although these differences were not statistically significant.

We also ran a series of dose–response logistic regression models to assess whether the number of spins earned through the incentive program predicted the likelihood of PrEP adherence at the 90-day follow-up. In the first model, the number of spins earned by the 30-day visit was significantly associated with PrEP adherence at 90 days (*B* = 1.04, *SE* = 0.27, *p* <.001, OR = 2.82, 95% CI [1.65, 4.82]). Each additional spin at 30-days nearly tripled the odds of being adherent at 90 days, and the model explained 28% of the variance in adherence (Nagelkerke R^2^ = 0.28). In a second model, total spins accrued over the 90-day period demonstrated an even stronger dose–response relationship (*B* = 1.54, *SE* = 0.35, *p* <.001, OR = 4.67, 95% CI [2.34, 9.32]), accounting for 75% of the variance in adherence Proportions of participants who demonstrated PrEP uptake, sustained use, and adherence are calculated based on the number of participants who received a PrEP prescription (denominator). Standard condition includes participants enrolled from 8/1/22 to 4/14/23 (37 weeks). Identity-based behavioral economic condition includes participants enrolled from 4/15/23 to 12/31/23 (37 weeks) (Nagelkerke R^2^ = 0.75). Each additional total spin increased the odds of adherence to PrEP at 90 days by approximately 4.7-fold. Model fit was excellent for both analyses (Hosmer-Lemeshow *p*s > 0.30), and predictive accuracy was high (overall classification rates = 71% and 92% for the 30-day and total-spin models, respectively). Together, these results indicate a clear and graded dose–response pattern, such that greater engagement with the spin-based incentive program, both early in participation and cumulatively, was strongly associated with subsequent PrEP adherence at 90 days.

### Maintenance (Costs)

All participants received $225 in study payments as incentives for participating in the three study visits. For the 72 participants in the behavioral economic incentives condition, they earned a total of 242 spins (average of 3.36 total spins per participant across visits). The average cost per spin was $15.18, and the average incremental intervention cost per participant was $51.02. The total cost of all prizes distributed was $3,673.76.

## Discussion

This pilot study demonstrated that integrating identity-based behavioral economic incentives into a community-led PrEP navigation program was both acceptable and feasible, and significantly improved adherence to PrEP at 90 days. Adherence rates were 22.3% higher in the behavioral economic incentive condition compared to the standard *PrEP Well* program, representing a moderate effect size. Participants who earned more spins also had higher odds of being adherent to PrEP. Importantly, this improvement was achieved at an average additional cost of $51.02 per participant, which is comparable to or lower than financial incentives used in other HIV prevention trials [22, 24]. The use of incentives that were not only appealing but also affirming of TGNB identities may have contributed to our promising outcomes. Providing prizes chosen, endorsed, and sourced by members of the TGNB community offered participants a sense of being seen and valued, which may be particularly impactful for TGNB individuals who often face systemic discrimination and exclusion from health care. The community-driven design of our behavioral economic enhancements is a major strength of the study and underscores the importance of tailoring HIV prevention strategies for marginalized populations.

These findings extend a growing literature applying behavioral economic incentives to PrEP engagement. Prior incentive-based PrEP studies have generally reported mixed and often modest effects, with benefits that vary by incentive type, magnitude, and population, and with relatively few interventions tailored to the structural realities of marginalized groups [19–24]. Reviews of financial and lottery-based incentives suggest that they can support PrEP-related behaviors but that effects are often inconsistent and rarely sustained [22, 23]. Against this backdrop, the moderate, dose-dependent improvement in 90-day adherence observed here is notable and suggests that embedding incentives within a community-led, identity-affirming program, rather than delivering financial incentives alone, may help account for the stronger engagement we observed. By tailoring both the target behaviors and the rewards to TGNB community priorities, our approach addresses a gap in prior work, which has seldom centered the identities and lived realities of the populations most affected by HIV inequities.

The study also illustrates how behavioral economic principles can be creatively applied to HIV prevention. The prize wheel leveraged multiple motivational mechanisms, e.g., uncertainty [44], immediacy [45], and positive reinforcement [46] from peer navigators, to make PrEP engagement both rewarding and fun. The clear and graded dose–response relationship found in the logistic regression models reinforces that the behavioral mechanism of the incentive program operated as intended; greater engagement with the wheel translated directly into higher PrEP adherence. Qualitative data suggested that participants valued prizes they would not typically buy for themselves, describing them as “treats” that elevated the experience. For example, one participant noted that receiving Pokémon cards, something they were unable to have as a child, felt especially meaningful. Interestingly, participants did not appear highly sensitive to actual prize cost, as inexpensive items like scented candles and fidget spinners were among the most popular. Together, these findings suggest that both the psychological experience of the incentive process and the behavioral dose (number of spins) contributed meaningfully to PrEP adherence gains at 90 days. This finding suggests that high perceived value can be achieved with low-cost items, offering strategies to enhance cost-effectiveness in future implementation trials. At the same time, high-demand items such as Bluetooth speakers and headphones consistently emerged as top-tier choices, suggesting that maintaining a mix of higher and lower value, practical, fun, and identity-affirming items is ideal. Notably, blankets emerged as one of the most frequently selected items, a pattern likely driven by the housing instability and precarious living situations experienced by many participants. This underscores the importance of tailoring incentive menus to the unique needs and realities of the target population, ensuring that items are not only engaging but materially responsive to participants’ day-to-day challenges.

The implementation of identity-based incentives within *PrEP Well* can be understood through implementation science frameworks. The EPIS [34] model guided our process, with early phases focused on community engagement, participatory prize selection, and collaboration with TGNB-owned businesses. Implementation required adaptations such as inventory systems and a prize “lookbook” to streamline administration, while sustainment considerations included balancing cost with authenticity and community alignment. In addition, outcomes were evaluated using the RE-AIM framework [47, 48]. The incentives improved *effectiveness* by increasing PrEP adherence, demonstrated *acceptability* and *feasibility* among participants and staff during *implementation* within the community center’s workflows. *Maintenance* will depend on future resources, scalability, and ongoing refinement of prize selection based on participant feedback, informed by the cost data collected in the current trial. Future implementation studies could further leverage the dose-response findings to optimize reinforcement schedules, testing whether fewer or differently timed opportunities to earn spins could maintain comparable adherence outcomes while reducing costs and administrative demands.

### Limitations and Future Directions

Several limitations must be acknowledged. First, maintaining an inventory of identity-based incentives required additional staff time, space, and resources beyond the direct costs of prizes. While the “lookbook” streamlined prize selection and minimized display needs, inventory management remained labor-intensive. Second, bulk purchasing helped reduce costs, but unpopular items risked remaining in stock, creating inefficiencies. Third, while working with TGNB-owned businesses was highly rewarding and produced high-quality items, this process required more time and financial investment than sourcing from large retailers. Future research should explore strategies for scaling these approaches, such as establishing standing partnerships with TGNB-owned businesses, streamlining sourcing systems, or testing narrower ranges of prize costs to reduce overall intervention costs while maintaining participant satisfaction. In addition, replication studies should evaluate whether the strong dose–response association observed here persists over longer follow-up periods and whether cumulative engagement predicts sustained PrEP use beyond 90 days. Finally, as a non-randomized pilot study, findings must be interpreted cautiously, though the phased design and baseline comparability across conditions increase confidence in the observed effects. Because the two conditions were enrolled sequentially rather than concurrently, secular trends, history effects, or unmeasured differences in who enrolled during each phase may have contributed to the observed differences, and selection effects cannot be fully ruled out. In addition, all participants were recruited from a single trans-led community center in Los Angeles, which may limit the generalizability of findings to TGNB populations in other regions, service settings, or program contexts.

## Conclusions

Findings from this pilot suggest that identity-based behavioral economic incentives can be successfully integrated into community-based, TGNB-led service delivery models to enhance PrEP engagement and adherence. Beyond their monetary value, the incentives’ alignment with TGNB identities likely contributed to their impact by fostering authenticity, trust, and a sense of community ownership. Taken together, the results support the idea that behavioral reinforcement strategies can operate in both psychological (motivation, engagement) and quantitative (dose) dimensions to sustain health-promoting behaviors. Scaling such interventions could help reduce persistent disparities in HIV prevention outcomes among TGNB adults. Future studies should test the long-term effects, scalability, and cost-effectiveness of identity-based incentives in larger and more diverse samples.

## Figures and Tables

**Table 1:** *PrEP Well* behavioral tasks leading to spin opportunities From: Identity-Based Behavioral Economic Incentives Increased PrEP Adherence for Transgender and Nonbinary Adults in a Trans-Led PrEP Navigation Program

Behavioral Task	Study Visit	Number of Spins
Attended Information Session about PrEP Well	Baseline	1 Spin
Welcome to *PrEP Well* Spin	Baseline	1 Spin
Attend Initial PrEP Appointment with PrEP Provider	30 or 90	1 Spin
Filled PrEP Rx at Pharmacy	30 or 90	1 Spin
Attended Lab/STI Testing Visi	30 or 90	1 Spin
Urine Test Shows PrEP Adherence	30 or 90	1 Spin
Congrats on Finishing PrEP Well Program	90	1 Spin
Discretionary Spin for other activities or attempts	Any	1 Spin

Max 3 Spins on Any visit

**Table 2:** *PrEP Well* participant demographics comparing standard and behavioral enhancement conditions From: Identity-Based Behavioral Economic Incentives Increased PrEP Adherence for Transgender and Nonbinary Adults in a Trans-Led PrEP Navigation Program

Characteristic	All Participants *N* = 108 *N* (%)	Standard PrEP Well Program *n* = 36 *N* (%)	Identity-Based Behavioral Economic Enhancements *n* = 72 *N* (%)	Test for Differences by Condition Statistic, *p*-value
Age, mean (SD)	23.9 (9.8)	24.3(4.6)	23.9(11.1)	*t*(106) = 0.21, *p* =.84
Gender Identity				*X*^2^(2) = 2.36, *p* = .31
Trans woman	60(55.6)	23(63.9)	37(51.4)	
Trans man	24(22.2)	8(22.2)	16(22.2)	
Nonbinary and other gender-expansive identities (e.g., genderqueer)	24(22.2)	5(13.9)	19(26.4)	
Assigned sex at birth				*X*^2^(2) = 1.34, *p* =.51
Male	78 (72.2)	24(66.7)	54(75.0)	
Female	28(25.9)	12(33.3)	16(22.2)	
Intersex	2(1.9)	0(0.0)	2(2.8)	
Race/ethnicity				*X*^2^(5) = 0.84, *p* =.93
Native American or Alaska Native	5(4.6)	2(5.6)	3(4.2)	
Black or African American	15(13.9)	4(11.1)	11(15.3)	
Asian/Pacific Islander	6(5.6)	0(0.0)	6(8.3)	
White	17(15.7)	7(19.4)	10(13.9)	
Hispanic or Latinx	49(45.4)	17(47.2)	32(44.4)	
Multi-racial/other	16(14.8)	6(16.7)	10(13.9)	
Sexual orientation				*X*^2^(3) = 1.79, *p* =.62
Gay or lesbian	5(4.6)	1(2.8)	4(5.6)	
Bisexual or pansexual	29(26.9)	9(25.0)	20(27.8)	
Straight	46(42.6)	14(38.9)	32(44.4)	
Other queer or questioning identities	28(25.9)	12(33.3)	16(22.2)	
Highest level of education				*X*^2^(4) = 3.27,p =.51
Some high school	8(7.4)	2(5.6)	6(8.3)	
High school (includes GED)	45(41.7)	19(52.8)	26(36.1)	
Associate’s, technical degree, or some college	29(26.9)	7(19.4)	22(30.6)	
College graduate	21(19.4)	6(16.7)	15(20.8)	
More than bachelor’s degree	5(4.6)	2(5.6)	3(4.2)	

**Table 3:** Stage of PrEP continuum reached by study condition From: Identity-Based Behavioral Economic Incentives Increased PrEP Adherence for Transgender and Nonbinary Adults in a Trans-Led PrEP Navigation Program

Participant Outcomes	All Participants *N* (%)	Standard PrEP Well Program *n* (%)	Identity-Based Behavioral Economic Enhancements *n* (%)	Test for Differences by Condition X^2^ statistic, *p*-value, ϕ-value
Enrolled in *PrEP Well* (received peer outreach/PrEP education, HIV testing, and peer navigation).	*N* = 108	*n* = 36	*n* = 72	
Received PrEP prescription (attended PrEP visit with provider, labs provided, and PrEP prescription written)	58(53.7)	16(44.4)	42(58.3)	*X*^2^(1,108) = 1.86,p = .17, ϕ = 0.13
PrEP uptake (confirmed via urinalysis at 30-day follow-up visit)	52(89.7)	14(87.5)	38(90.5)	*X*^2^ (1,108) = 1.85, *p* = .17, ϕ = 0.13
Sustained PrEP use (confirmed via urinalysis at 9O-day follow-up visit)	43(74.1)	10(62.5)	33(78.6)	*X*^2^(1,108) = 3.27, *p* = .07, ϕ = 0.17
PrEP adherence (confirmed via dried blood spot analysis at 9O-day follow-up visit)	42(72.4)	9(56.3)	33(78.6)	*X*^2^(1,108) = 4.38, *p* < .05*, ϕ = 0.20

Proportions of participants who demonstrated PrEP uptake, sustained use, and adherence are calculated based on the number of participants who received a PrEP prescription (denominator). Standard condition includes participants enrolled from 8/1/22 to 4/14/23 (37 weeks). Identity-based behavioral economic condition includes participants enrolled from 4/15/23 to 12/31/23 (37 weeks)
